# New ideas in preventing castration-resistant prostate cancer

**DOI:** 10.18632/oncotarget.21396

**Published:** 2017-09-30

**Authors:** Yuan-Chin Tsai, Yen-Nien Liu

**Affiliations:** Yen-Nien Liu: Graduate Institute of Cancer Biology and Drug Discovery, College of Medical Science and Technology, Taipei Medical University, Taipei, Taiwan

**Keywords:** transforming growth factor induced, androgen-deprivation therapy, castration-resistant prostate cancer, epithelial-to-mesenchymal transition, integrin

Prostate cancer is the third leading cause of cancer death in American men. The rationale behind the therapeutic approaches that manage this disease for decades is mainly based on the fundamental works by Charles Huggins, the Nobel Laureate in medicine, revealing the androgen-dependent nature of prostate cancer. Thus, hormone therapy was implemented in the clinical setting and androgen-deprivation therapy (ADT) has been one key regimen for patients with recurrence after localized treatments, such as prostatectomy. However, resistance to ADT eventually occurs and leads to castration-resistant prostate cancer (CRPC). Studies of this progression found that cancer cells can utilize multiple mechanisms to overcome the castrated level of testosterone, including altered expression/mutations in the androgen receptor (AR) or intratumoural biosynthesis of its agonist (i.e. dihydrotestosterone) via perturbed steroid metabolism [1].

Since CRPC in part still relies on functions of the AR, novel therapeutics inhibiting either steroid metabolic enzymes or the AR have improved the survival rates in CRPC patients. However, none of those are curative, and resistance eventually develops [2], consistent with the repeated cycles of treatment-dormancy-relapse in managing prostate cancer [3]. To make the situation worse, recurrent prostate cancer becomes even more malignant and capable of spreading out to other areas in the patients. Thus, knowing the predictable outcomes following androgen/AR-targeted therapies, it seems reasonable to revisit roles of the AR during the progression. The epithelial-to-mesenchymal transition (EMT) is involved in many aspects of malignant progression (e.g. drug-resistance and metastasis), and several lines of evidence have shown that ADT promotes the EMT. In addition, it was shown that mice with epithelium-specific AR knockout exhibit increased proliferation in ventral prostate and further develop frequent metastasis when crossed with the strain containing SV40 T antigen oncogenes [4, 5]. Altogether, these results support that the AR exhibits anti-tumor effects and suppresses oncogenic programs.

**Figure 1:**
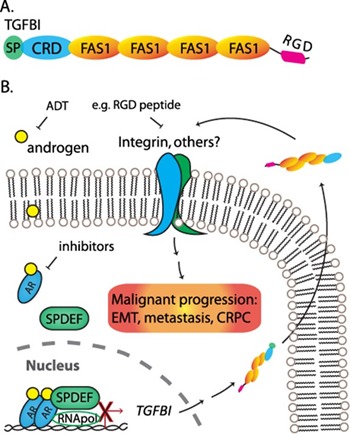
Working model of TGFBI-mediated malignant progression of prostate cancer **A.** Structure domains of TGFBI. SP: secretory signal peptide. CRD: cysteine-rich domain. FAS1: fasciclin domain. RGD: a RGD motif at the C-terminus. **B.** Androgen activates AR signaling and suppresses TGFBI expression through SPDEF-mediated downregulation. TGFBI secretes to extracellular space and interacts with membrane receptors, e.g. integrin, facilitating malignant progression.

Transforming growth factor β (TGFβ) is a master player of the EMT and implicated in promoting tumor metastasis. One of the downstream factors following TGFβ signaling is transforming growth factor induced (*TGFBI*), also known as βig-h3 or BIGH3, which was shown to be overexpressed in many types of cancer and to promote metastasis of colon cancer [6]. TGFBI is a secreted protein containing a signal peptide (SP) at N-terminus, followed by a cysteine-rich domain (CRD), four fasciclin domain (FAS1), and a C-terminal RGD motif (Figure 1A). The fasciclin domains are likely responsible for the cell adhesion functions of TGFBI and its RGD motif can associate with many types of integrins. In combination, secretion of TGFBI should facilitate all stages of a metastasis process, from intravasation to distal localization. Consistent with this mode of action, it was shown that TGFBI-promoted metastasis is dependent on the RGD motif and integrin α_v_β_5_ [6].

Chen and coworkers identified TGFBI as an EMT marker that is positively regulated by TGFβ signaling in prostate cancer [7]. Following downregulation using small interfering RNA approach, TGFBI was shown to be essential in metastasis of prostate cancer. Importantly, TGFBI is suppressed by AR signaling partly through a SPDEF-mediated function. SPDEF is an androgen-inducible factor and a member of the ETS transcription factor family. Chen et al. found that SPDEF transcriptionally represses TGFBI and that ADT-treated patients have reduced SPDEF and increased TGFBI. Based on these findings, a TGFBI-mediated molecular mechanism is proposed to explain the malignant progression following androgen/AR-targeted therapies (Figure 1B).

Adding the TGFBI in the picture, it immediately provides new therapeutic strategies to be evaluated in preventing, or even treating, metastatic CRPC. Novel agents targeting specific intergrins (e.g. α_v_β_5_) or competing with the RGD motif of TGFBI can be tested either after or in combination with androgen/AR-targeted therapies (Figure 1B). In addition, inventions capable of reducing *TGFBI* expression could potentially benefit another genetic disease, corneal dystrophy, which results from mutations in TGFBI leading to amyloid-like deposit in the eyes. However, it should be cautious for patients who are undergoing certain types of treatments, since TGFBI could be essential for the therapeutic effects of taxel-based chemotherapy [8]. Another application of TGFBI-targeted approach is in the treatment of neuroendocrine prostate cancer (NEPC), which is deficient of AR signaling. The role of TGFBI in NEPC requires further investigation.
